# Sustainability consciousness among nursing students in Egypt: a cross-sectional study

**DOI:** 10.1186/s12912-024-01990-1

**Published:** 2024-05-21

**Authors:** Marwa Ahmed El-Sayed Mohamed, Eman Ghallab, Ragaa Abdullah Ahmed Hassan, Shaimaa Mohamed Amin

**Affiliations:** 1 Community Health Nursing Department, Faculty of Nursing, Alexandria University, Alexandria, Egypt; 2 Nursing Education Department, Faculty of Nursing, Alexandria University, Alexandria, Egypt; 3 Family and Community Health Nursing Department, Faculty of Nursing, Sohag University, Sohag, Egypt; 4 Community Health Nursing Department, Faculty of Nursing, Damanhour University, Damanhour, Egypt

**Keywords:** Sustainability Development, Consciousness, Knowingness, Attitude, Behaviors, Environment, Egyptian Universities, Nursing Education, United Nations, UNESCO, Education for Sustainable Development, Sustainable Development Goals

## Abstract

**Background:**

Recognizing nurses as pivotal change agents and urgent preparation for the next generation is crucial for addressing sustainability issues and cultivating a sustainable healthcare system. Reports highlight gaps in the integration of sustainable development goals (SDGs) into nursing curricula, prompting the International Council of Nurses to stress the importance of sustainable development in nursing education. The extent to which nursing students are aware of sustainability issues remains to be determined. This study addresses a global and Egyptian research gap by evaluating and comparing Sustainability Consciousness (SC) levels among nursing students at three universities, providing insights into awareness, attitudes, and behaviors related to sustainability.

**Methods:**

This descriptive comparative study was conducted across three nursing faculties within three Egyptian public universities. The target population for this study was undergraduate nursing students enrolled in these universities during the academic year 2022-2023. This study used the extended version of the Sustainability Consciousness Questionnaire (SCQ-L) to measure individuals' knowingness, attitudes, and behaviors related to the three dimensions of sustainable development: environmental, social, and economic.

**Results:**

More than half of the nursing students across the three universities expressed unfamiliarity with the SDGs. Social media was the most common source of information across the three universities. Sohag University recorded the highest median (IQR) scores in sustainability knowingness (72.0, IQR: 70.0-81.0), attitudes (56.0, IQR: 53.0-61.5), and behavior (65.0, IQR: 60.0-69.0) across their respective dimensions, as well as in overall SC (195, IQR: 184.5-208.5). This was followed by Damanhour University, with a median score of 179.0 (IQR: 124.5-194), and then Alexandria University, with a median score of 116.0 (IQR: 85.5-153), all of which were significantly different (all with p values=0.000). Older students from rural areas exhibited higher SC median scores, while students with insufficient family income and no familiarity with the SDGs had lower SC scores.

**Conclusions:**

This study highlighted the prevalent reliance on social media for information among nursing students across these universities, emphasizing the pivotal role of academic institutions in integrating sustainability development concepts in nursing education. Sohag University's notable commitment to sustainability practices has contributed to the higher SC of its students compared to Alexandria and Damanhour Universities. The study also identified age, place of residence, family income, and familiarity with the SDGs as consistent predictors of sustainability consciousness.

**Supplementary Information:**

The online version contains supplementary material available at 10.1186/s12912-024-01990-1.

## Introduction

Humanity faces several sustainability issues, often resulting from human interaction and the environment [1]. Despite not being intentionally harmful, human behavior and lifestyle are significant contributors to these challenges, exacerbating environmental, economic, and social problems [2, 3]. Addressing these challenges requires fundamental changes in how people think and act, which can be achieved through education [4]. At the same time, global actions are required to promote sustainable development to combat such challenges [5]. Sustainable development has been defined as "development that meets the needs of the present without compromising the ability of future generations to meet their own needs" (World Commission on Environment and Development WCED, 1987) [6]. For sustainable development to be attained, it is necessary to harmonize its three fundamental components: "economic growth, social inclusion, and environmental protection" [7]. This calls for collaborative efforts toward building a safer, more resilient, and equitable future on Earth for everyone [8].

In 2015, the United Nations General Assembly adopted the 2030 Agenda for Sustainable Development, incorporating 17 Sustainable Development Goals (SDGs). These goals are designed to address the economic, social, and environmental dimensions of sustainable development. These goals comprehensively address the significant global challenges that endanger human and planet survival [9]. Education is critical to achieving these SDGs, as outlined in UNESCO reports from 2006, 2009, and 2014a. Individuals in all societies should possess the knowledge, skills, and attitudes that help them deal with sustainability challenges. The 2030 Agenda recognizes education as an independent goal (SDG 4) and includes educational indicators in other SDGs [10–12]. This positionsposition education as a primary catalyst for equipping individuals with the competencies and knowledge needed for a sustainable world. Since 1992, UNESCO has promoted Education for Sustainable Development (ESD) to empower learners of all ages to make informed choices and act sustainably for societal and environmental change [13].

In this context, higher education institutions play a critical role. They are expected to actively apply educational measures to equip graduates with sustainability competencies, which are essential for achieving the SDGs [14]. This form of education is designed to bring about changes in learners' knowledge, skills, and attitudes, cultivating a society that is more sustainable and equitable [15, 16]. Universities can educate students about sustainable development by integrating the concept into their education systems and curricula [17, 18]. One of the primary goals of universities should be to ensure that all students, regardless of their field of study, achieve a high level of sustainability awareness, contributing to the broader goal of sustainable development [19].

As sustainable development and ESD have evolved, a new concept, Sustainability Consciousness, has emerged. This concept was developed by a Swedish research group to measure the effect of the implementation of ESD on students at Swedish schools [20, 21]. Sustainability Consciousness can be simply defined as "the experience or awareness of sustainability phenomena" [20], including the individual's knowledge, actions, attitudes, and experiences that form their identity [22]. Thus, SC is a combination of knowledge, behaviors, and attitudes about sustainability [23]. It integrates the environmental, social, and economic dimensions of sustainable development, emphasizing the importance of knowingness, attitudes, and behaviors in these three dimensions [24].

Sustainability knowingness relates to an awareness of the theoretical aspects of sustainable development, while sustainability attitude refers to being concerned about sustainability that subsequently translates into sustainable behaviors. Sustainability behavior refers to actions that support and contribute to sustainable development. To measure SC, a questionnaire was developed to assess awareness, attitudes, or behaviors related to the environmental, social, or economic aspects of SD [23, 25]. These dimensions are essential in evaluating a person's SC [26]. This is especially significant in fields such as healthcare, where SC directly impacts professional practices.

The healthcare sector, including nursing, has a clear mandate to fulfill the United Nations' SDGs. This mainly includes a commitment to achieve the goal of ensuring healthy lives and promoting the well-being of individuals, regardless of age [27]. Nurses are pivotal in responding to sustainability issues, as they are change agents capable of improving public health and managing health resources effectively [28, 29]. There is a pressing need to prepare future nurses to address these issues and actively contribute to building a sustainable healthcare system [30]. This preparation is crucial to help them understand the impact of sustainability issues on public health, provide environmental health education, and offer informed counseling [31–33]. The International Council of Nurses (ICN) emphasizes that sustainable development should be part of nursing curricula and continuing education, aiming to empower nurses to assume leadership roles in fostering sustainable practices within healthcare organizations and lead initiatives in sustainable development in healthcare [29].

Despite these imperatives, reports indicate existing gaps in addressing sustainability issues, including the SDGs, within nursing curricula. The extent to which nursing students become aware of sustainability remains to be determined [15, 34]. While some studies have explored sustainability awareness among university students globally, more research needs to investigate and measure the SC of nursing students specifically, both on a global scale and within Egypt [32, 35–38]. Consequently, further research is essential to gain a deeper understanding of the SC of nursing students. Such insights will inform policies and practices and contribute to effectively preparing and shaping the future leaders of the nursing profession in the realm of sustainability.

In addition, previous research indicates that ESD varies according to cultural context, highlighting the need for more cross-cultural studies on diverse cultural perspectives and engagement with sustainable development [38, 39]. This study seeks to fill this gap by conducting a comparative analysis of SC, encompassing awareness, attitudes, and behaviors, among nursing students at three distinct Egyptian public universities, each situated in culturally unique regions. This research is the first to explore the SC of nursing students across different universities in Egypt.

## Methods

### Aim

This study aimed to assess and compare the levels of sustainability consciousness, encompassing knowingness, attitudes, and behaviors, among nursing students across three Egyptian public universities. Moreover, this study aimed to investigate the predictors of SC among students at these universities.

### Study design

This study utilized a descriptive comparative cross-sectional research design. This study adhered to the Strengthening the Reporting of Observational Studies in Epidemiology (STROBE) guidelines.

### Setting

The study occurred in three nursing faculties within three Egyptian public universities in Alexandria, Damanhour, and Sohag. These universities were selected based on several criteria, including their high student enrollment during the 2022-2023 academic year, the diverse backgrounds of students hailing from both rural and urban areas, and the representation of students from both Lower and Upper Egypt. Lower Egypt and Upper Egypt are historical and geographical divisions of ancient Egypt. Lower Egypt refers to the northern region of Egypt, which is adjacent to the Mediterranean Sea and encompasses the Nile Delta. Conversely, Upper Egypt denotes the southern part of the country, situated further upstream along the Nile River. Each region has a unique culture, tradition, and religious significance. Alexandria, the second largest city in Egypt, is situated on the Mediterranean coast and lies at the western edge of the Nile River Delta. Damanhour, located in Lower Egypt, serves as the capital of El-Beheira Governorate and occupies a central position within the western Nile Delta. Sohag, positioned in Upper Egypt, rests on the west bank of the Nile River. The reason for selecting universities from Upper and Lower Egypt was to capture a diverse range of perspectives and experiences, thereby reducing the potential for bias from focusing solely on one region or demographic group.

### Sampling and study population

The target population for this study was undergraduate nursing students enrolled in three Egyptian public universities during the 2022-2023 academic year. G power was employed to calculate the sample size considering a total population of 7554, a significance level of 0.05, a moderate effect size, a power of 0.80, and a 95% confidence interval. The minimum required sample size for each university was 390, which was rounded to 400 to compensate for possible non-response. Consequently, the total sample size was 1200 students, employing an equal allocation method that selected 100 students from each academic year using systematic random sampling. All students enrolled in the first through fourth levels were eligible to participate in the study.

### Outcome measurement instrument

#### Sustainability Consciousness

The extended version of the Sustainability Consciousness Questionnaire (SCQ-L) developed by Gericke et al. 2019 was used in this study [20]. The questionnaire was developed to measure individuals' knowledge, attitudes, and behaviors related to the environmental, social, and economic dimensions of sustainable development. The SCQ-L comprises 49 items, each rated on a five-point Likert scale ranging from "strongly disagree" (1) to "strongly agree" (5), with "disagree" as 2, "neutral" as 3, and "agree" as 4. It is structured into three sections, with the items in each section corresponding to the three critical dimensions of sustainable development: environmental, social, and economic. The first section, the "Sustainability Knowingness Scale," consists of 18 items distributed across three dimensions: environmental (6 items), social (8 items), and economic (4 items). The second section, the "Sustainability Attitudes Scale," encompasses 14 items categorized into three dimensions: environmental (4 items), social (6 items), and economic (4 items). In the third section, the "Sustainability Behavior Scale," there are 17 items divided into three dimensions: environmental (7 items), social (6 items), and economic (4 items).

The items on sustainability knowingness, attitude, and behavior cover what people acknowledge as essential components of sustainability, feelings, attitudes toward sustainable development and sustainability issues, and people's actions and behaviors. The instrument's psychometric properties were assessed by Gericke et al. in 2019, who reported that it is a reliable and valid tool, with Cronbach's alpha coefficients of α = 0.82, 0.73, and 0.79 for the sustainability knowingness scale, sustainability attitudes scale, and sustainability behavior scale, respectively.

### Data collection

The data were collected from September 2022 to December 2022. The SCQ-L questionnaire was distributed among students in various locations, including lecture rooms and libraries, from Saturday to Thursday between 9 am and 2 pm. The questionnaire was distributed to 1236 students to address incomplete or missing data. For each participant, completing the questionnaire took approximately 15-20 minutes. Additionally, demographic information, such as age, gender, academic year, place of residence, family income, and marital status, was collected using a demographic characteristics information form.

### Data analysis

SPSS 20.0 software was used to perform descriptive and inferential statistical analyses. Demographic data were summarized using descriptive statistics and the chi-square test to identify significant differences between the three universities. Statistical significance *p*<0.05 was considered. Since the data did not follow a normal distribution, nonparametric tests such as the Kruskal–Wallis and Mann–Whitney tests were used to identify significant differences among the three universities. The sustainability consciousness domains were summarized using medians and interquartile ranges (IQRs). Multiple linear regression was utilized to identify predictors of SC among nursing students.

## Results

### Sociodemographic characteristics and sources of SDG information

**Fig 1:**
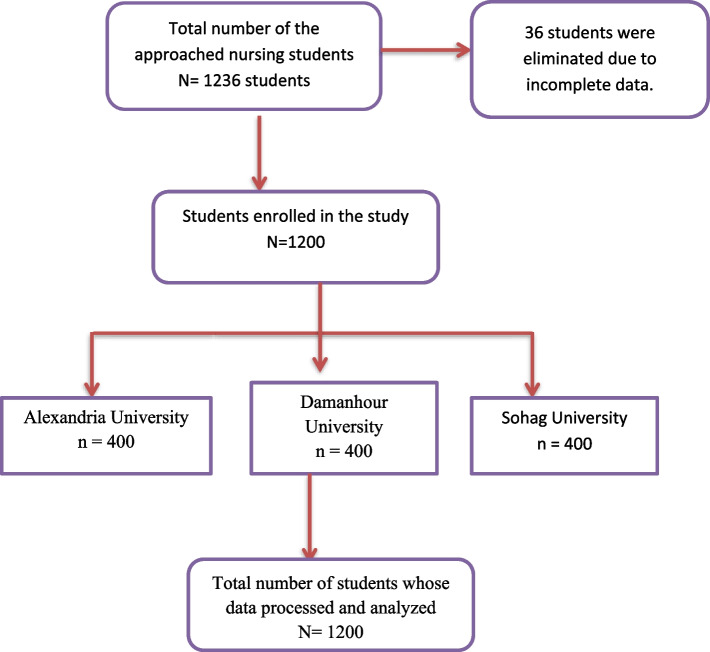
Participant recruitment flowchart

Of only 1236 student responses, 36 were eliminated due to incomplete data (i.e., failure to give consent, missing responses, and skipped question items). After the data were cleaned, a total of 1200 responses were analyzed (Fig. 1). Table 1 describes the demographic differences among Alexandria, Damanhour, and Sohag University students. There were significant differences between the three universities according to the students' sex, age, marital status, place of residence, family income (*p*=0.000), familiarity with the SDGs (*p*=0.002), and sources of information about the SDGs (*p*=0.000). First, there is a significant gender difference, with the university having a relatively balanced gender distribution, while Alexandria and Damanhour Universities have a predominance of female students. Second, there was a greater percentage of students aged 20 to less than 22 years in all three universities. Moreover, the marital status significantly differed, with most single students across the three universities.

**Table 1:** Distribution of Student Demographic Characteristics and Sources of SDG Information across Three Universities

**Items**	**Universities**			**Test of Significance (*p* value)**
	**Sohag**	**Damanhour**	**Alexandria**	
	**(*N*=400)**	**(*N*=400)**	**(*N*=400)**	
	No. (**%**)	No. (**%**)	No. (**%**)	
**Sex**				**χ**^**2**^**=44.8**
Male	204 (51.0)	118 (29.5)	133 (33.2)	(*p*=0.000*)
Female	196 (49.0)	282 (70.5)	267 (66.8)	
**Age (years)**				**χ**^**2**^_**6**_**=109.5**
<20	110 (27.5)	69 (17.2)	147 (36.8)	(*p*=0.000*)
20-<22	160 (40.0)	248 (62.0)	205 (51.2)	
22-<24	125 (31.2)	73 (18.2)	32 (8.0)	
≥ 24	5 (1.2)	10 (2.5)	16 (4.0)	
**Marital status**				**χ**^**2**^**=33.7**
Single	391 (97.8)	357 (89.2)	387 (96.8)	(*p*=0.000*)
Married	9 (2.2)	43 (10.8)	13 (3.2)	
**Place of residence**				**χ**^**2**^**=68.3**
Urban	116 (29.0)	105 (26.2)	207 (51.8)	(*p*=0.000*)
Rural	284 (71.0)	295 (73.8)	193 (48.2)	
**Family Income**				**χ**^**2**^**=28.9**
Sufficient	265 (66.2)	228 (57.0)	300 (75.0)	(*p*=0.000*)
Insufficient	135 (33.8)	172 (43.0)	100 (25.0)	
**Familiarity with SDGs**				
Yes	133 (33.2)	166 (41.5)	182 (45.5)	**χ**^**2**^**=12.9**
No	267 (66.8)	234 (58.5)	218 (54.5)	(p=0.002*)
**Sources of information about SDGs:**	***N*= (133)**	***N*= (166)**	**N= (182)**	**χ**^**2**^_**6**_**=109.5**
Academic courses	18 (13.5)	34 (20.5)	21 (11.5)	(p=0.000*)
Books	0 (0.0)	9 (5.4)	15 (8.2)	
Mass media	15 (11.3)	18 (11.0)	22 (12.1)	
Social Media	66 (49.6)	99 (59.6)	114 (62.6)	
Websites	27 (20.3)	1 (0.6)	0 (0.0)	
Friends and family	7 (5.3)	3 (1.8)	10 (5.5)	
Training courses	0 (0.0)	2 (1.2)	0 (0.0)	

**χ**^2^ = Chi square test

^*^Significant at *p*≤0.05

Regarding the place of residence, Alexandria University has a greater percentage of students living in urban areas than Sohag and Damanhour. Most students at Alexandria University reported sufficient income compared to those at Damanhour and Sohag. Familiarity with the SDGs also shows significant variations, with more students at Alexandria University indicating familiarity (45.5%). Moreover, sources of information about the SDGs varied significantly among the three universities, highlighting the prevalence of social media as the most common source across the three universities, with Alexandria having the highest percentage (62.6%).

### Comparison of the sustainability dimensions and constructs among students at the three universities

The Kruskal‒Wallis test revealed significant variations among the three universities in terms of sustainability knowingness (H=348.72, *p*=0.000), sustainability attitudes (H=380.12, *p*=0.000), and sustainability behavior (H=500.91, *p*=0.000) across the environmental, social, and economic dimensions within each of them (all with p values=0.000). Additionally, differences were observed in the overall SC (H=782.49, *p*=0.000). Sohag University consistently recorded the highest median (IQR) scores for sustainability knowingness (72.0, IQR: 70.0-81.0), attitudes (56.0, IQR: 53.0-61.5), and behavior (65.0, IQR: 60.0-69.0) across their respective dimensions, as well as overall SC (195, IQR: 184.5-208.5), followed by Damanhour and then Alexandria University (Table 2).

**Table 2:** Comparison of Sustainability Dimensions and Constructs among Students at Three Universities

**Sustainability dimensions and constructs**	**Universities**			**Test of significance** **(*p* value)**
	**Alexandria** **(*N*=400)**	**Damanhour** **(*N*=400)**	**Sohag** **(*N*=400)**	
	**Median (IQR)**	**Median (IQR)**	**Median (IQR)**	
**Sustainability Knowingness (Total)**	**43.0(30.0-62.0)**	**69.0(47.0-74.0)**	**72.0(70.0-81.0)**	**H=348.72** (*p*=0.000*)
• Environmental	14.0(9.0-18.0)	21.0(15.0-23.0)	23.0(22.0-26.0)	H=382.46 (*p*=0.000*)
• Social	20.0(12.0-28.0)	32.0(20.0-34.5)	33.0(32.0-38.0)	H=291.79 (*p*=0.000*)
• Economic	8.0(4.0-16.0)	16.0(10.0-17.5)	16.0(16.0-19.0)	H=247.75 (*p*=0.000*)
**Sustainability Attitudes (Total)**	**34.0(23.0-46.0)**	**52.0(36.0-56.0)**	**56.0(53.0-61.5)**	**H=380.12** (*p*=0.000*)
• Environmental	8.0(5.0-12.0)	13.0(10.0-15.0)	16.0(14.0-17.0)	H=406.22 (*p*=0.000*)
• Social	14.0(8.0-22.0)	23.0(15.0-25.0)	24.0(23.0-27.0)	H=297.02 (*p*=0.000*)
• Economic	12.0(6.0-16.0)	16.0(10.0-17.5)	16.0(16.0-19.0)	H=259.31 (*p*=0.000*)
**Sustainability Behavior(Total)**	**37.0(28.0-49.0)**	**57.0(44.0-64.0)**	**65.0(60.0-69.0)**	**H=500.91** (*p*=0.000*)
• Environmental	14.0(11.0-19.0)	23.0(18.0-26.0)	27.0(24.0-28.0)	H=517.71 (*p*=0.000*)
• Social	14.0(9.0-20.0)	21.0(16.0-24.0)	24.0(21.0-25.0)	H=329.35 (*p*=0.000*)
• Economic	8.0(6.0-12.0)	13.0(10.0-15.0)	15.0(13.0-16.0)	H=422.77 (*p*=0.000*)
**Sustainability Consciousness (Total)**	**116.0(85.5-153)**	**179.0(124.5-194)**	**195(184.5-208.5)**	**H=782.49** (*p*=0.000*)

IQR: Interquartile Range** (**Q1-Q3) (First quartile – Third quartile)

**H:** H for the Kruskal‒Wallis test*: Statistically significant at *p* ≤ 0.05

Regarding the sustainability constructs, sustainability knowingness obtained the highest median scores among students at the three universities, followed by sustainability behavior, with sustainability attitudes registering the lowest median scores.

Within the knowingness construct, the social dimension achieved the highest median score across all three universities, followed by the environmental and economic dimensions. Concerning sustainability attitudes, the social dimension once again secured the highest median scores among students at all universities, followed by the economic dimension, with the environmental dimension registering its lowest scores. Regarding sustainability behavior, the environmental dimension obtained the highest median scores, closely followed by the social dimension, while the economic dimension recorded the lowest median scores among students across the three universities.

### Associations between Demographic Characteristics and Sustainability Consciousness

Table 3 shows statistically significant differences in SC based on various demographics. Although there was no significant difference in SC between males and females, age emerged as a significant factor (H= 24.65, *p* =0.000); students aged 22-<24 years obtained the highest median consciousness scores, while those aged ≥24 years obtained the lowest scores. The academic year also had a significant impact (H =18.45, *p*= 0.000), revealing that second-year students exhibited the highest consciousness scores.

**Table 3:** Association between student demographic characteristics and sustainability consciousness

**Items**	**Total Sustainability Consciousness**
	**Median (IQR)**
**Sex**	
Male	177.0(116.0-197.0)
Female	178.0(120.0-195.0)
**Test of significance (p value)**	**Z= 0.430 (p=0.667)**
**Age (years)**	
<20	167.5(115.0-196.0)
20-<22	177.0(113.0-194.0)
22-<24	187.0(153.0-200.0)
≥ 24	153.0(109.0-1870)
**Test of significance (p value)**	H=24.65 (p=0.000*)
**Academic year**	
First	163.5(108.5-195.0)
Second	183.5(146.0-197.5)
Third	165.5(111.5-196.0)
Fourth	180.0(131.5-196.0)
**Test of significance (p)**	H=18.45 (p=0.000*)
**Marital status**	
Single	176.0(117.0-196.0)
Married	184.0(164.0-193.0)
**Test of significance (p value)**	**Z= 1.349 (p=0.177)**
**Place of residence**	
Urban	165.5(110.0-194.0)
Rural	181.0(125.5-197.0)
**Test of significance (p value)**	Z= 3.623 (p=0.000*)
**Family Income**	
Sufficient	181.0(131.0-197.0)
Insufficient	162.0(111.0-192.5)
**Test of significance (p value)**	Z= 3.866 (p=0.000*)
**Familiarity with SDGs**	
Yes	182.0(138.0-198.0)
No	173.0(112.0-194.5)
**Test of Significance (p value)**	Z= 3.234 (p=0.001*)

*IQR* Interquartile Range (Q1-Q3) (First quartile – Third quartile)

H:H for the Kruskal‒Wallis test, Z: Z for the Mann‒Whitney U test

^*^Significant at *p*≤0.05

Additionally, a significant difference in SC was found between rural and urban students (Z=3.623, *p*=0.000), demonstrating that rural students had greater SC. Family income and SDG familiarity (Z= 3.866, *p*=0.000 and Z=3.234, *p*=0.001, respectively) also contributed significantly to SC. Students from families with sufficient income and those familiar with the SDGs demonstrated higher levels of consciousness.

### Predictors of sustainability consciousness among nursing students

The findings of the multiple linear regression analysis revealed different patterns in the predictors of SC among nursing students across the three universities (Table 4).

**Table 4:** Multiple linear regression to identify predictors of sustainable consciousness among nursing students

**Variables**	**Alexandria** **University (*N=*400)**		**Damanhour University (*N*=400)**		**Sohag** **University** **(*N*=400)**	**p**	**Total sample (*n* = 1200)**	**p**
	**B** **(95% CI)**	**p**	**B** **(95% CI)**	**p**	**B (95% CI)**		**B** **(95% CI)**	
Students' age (>24)	-1.635 (-5.295 - 2.026)	0.381	-2.659^*^ (-5.018- -0.300)	0.027^*^	0.099 (-1.958- 2.155)	0.925	3.228^*^ (1.109- 5.346)	0.003^*^
Students' sex (female)	-0.552 (-4.314- 3.210)	0.773	7.204^*^ (4.386 - 10.021)	=0.000^*^	-1.007 (-2.900- 0.886)	0.296	-0.886 (-3.175- 1.404)	0.448
Academic year (Fourth)	0.502 (-1.868- 2.872)	0.677	2.056^*^ (0.638 - 3.473)	0.005^*^	0.113 (-1.339- 1.565)	0.879	-0.966 (-2.378- 0.447)	0.180
Place of residence (Rural)	-3.342 (-6.925- 0.240)	0.067	-1.253 (-4.074- 1.568)	0.383	1.750 (-0.350- 3.850)	0.102	5.059^*^ (2.742 - 7.376)	=0.000^*^
Family Income (Insufficient)	3.823 (-0.288- 7.934)	0.068	-17.065^*^ (-19.816- -14.314)	=0.000^*^	0.455 (-1.550- 2.460)	0.656	-4.262^*^ (-6.654 - -1.869)	=0.000^*^
Marital status (Married)	0.231 (-10.253- 10.716)	0.965	0.112 (-4.009 - 4.232)	0.958	-0.223 (-6.566- 6.119)	0.945	2.300 (-2.669 - 7.268)	0.364
Familiarity with SDGs (No)	-3.559 (-7.156- 0.038)	0.052	-5.852^*^ (-8.581- -3.124)	=0.000^*^	-2.839* (-4.833- -0.845)	0.005*	-2.971* (-5.270- -0.672)	0.011^*^
**R**^**2**^	**0.025**		**0.451**		**0.012**		**0.035**	

R^2^: Coefficient of determination

*B* Unstandardized Coefficients

*LL* Lower limit, UL: Upper Limit

^*^Statistically significant at *p* ≤ 0.05

At Alexandria University, none of the predictor variables demonstrated significant associations with SC. However, at the university, age, sex, academic year, family income, and familiarity with the SDGs emerged as significant predictors. Specifically, being female strongly predicted higher SC (*p*=0.000), while having no familiarity with the SDGs and insufficient family income were robust predictors of lower SC (p=0.000). Furthermore, being in their fourth academic year was associated with greater consciousness (*p*=0.005). At university, familiarity with the SDGs was a significant predictor, with students lacking this familiarity displaying lower SC.

When considering the total sample, which included students from all three universities, the predictors were consistent with those found in Damanhour and Sohag universities. Age, place of residence, family income, and familiarity with the SDGs all emerged as significant predictors of SC. Older students from rural areas exhibited greater consciousness, while students with insufficient family income and no familiarity with the SDGs displayed lower SC. Notably, these predictors collectively explain only a tiny proportion of the variance in SC, accounting for 3.5% (R2=0.035) of the observed differences.

## Discussion

The primary purpose of this study was to investigate the level of SC among nursing students across three Egyptian universities: Alexandria and Damanhour in Lower Egypt and Sohag in Upper Egypt. Interestingly, the findings of the present study highlighted that more than half of the nursing students across these universities expressed a lack of familiarity with the SDGs. Although this finding differs from that of Zainordin et al. (2017), who showed that 90% of students in their study were familiar with the SDGs [40], it is consistent with previous studies that revealed alarmingly low awareness levels regarding SDGs [35–37, 41].

The findings also revealed that across the three universities, students primarily relied on social media for information about the SDGs. Mass media, academic courses, and websites were used as secondary sources. This finding aligns with earlier studies on sustainability issues, indicating that the internet, mainly through social media platforms, stands as the predominant channel for acquiring knowledge among university students in Egypt [37, 42], Malaysia [43, 44], and even among educators in Italy [45]. Although the prominence of social media as a source for acquiring knowledge about sustainability issues might not be surprising, it raises concerns about the inadequate coverage of sustainability issues in academic settings [46, 47].

In the current study, sustainability knowingness emerged as the dominant construct, with students achieving the highest median scores across the three universities, closely followed by sustainability behavior, while sustainability attitudes received the lowest median scores. The findings indicate that while students fully grasp sustainability concepts, their attitudes toward sustainability in all dimensions need to be further developed. This finding is not unique to this study; similar trends have been identified in studies conducted across different countries, indicating that even when students possess good sustainability knowledge and behaviors, their attitudes may not be proportionally affected [36, 37, 48, 49]. This predominance of knowledge may be attributed to the fact that while social media, the primary information source for the students in the current study, allows easy access to factual information, contributing to their knowledge, it may not inherently promote the development of attitudes. Social media content tends to offer brief, isolated information designed for quick consumption and needs more context. Moreover, students may passively scroll through content without actively engaging in in-depth discussions and debates related to the SDGs necessary for attitude development toward sustainability.

Furthermore, the findings of the present study consistently revealed that the social dimension had the highest score for sustainability knowingness, followed by the environmental dimension, with the economic dimension consistently receiving the lowest score across all three universities. This finding aligns with a study conducted by Marcos-Merino et al. (2020), who found that Spanish students tended to allocate the highest scores to their sustainability knowledge in the social dimension [50]. Similarly, the students in the present study displayed more favorable attitudes toward social sustainability than toward other dimensions, supporting the observations of Ebrahim et al. (2022) [37], El-Hamed et al., 2022 [51], and Marcos-Merino et al. (2020) [50]. The students exhibited greater knowledge and held more positive attitudes toward social sustainability due to the widespread exposure to information and discussions on social sustainability issues such as poverty, social inequalities, and social justice. These topics frequently receive significant attention in Egypt's media and public discourse.

The nursing curriculum in the three universities under study also strongly emphasized social sustainability issues. Academic exposure to these issues likely enhanced understanding and cultivated positive attitudes toward social sustainability. Finally, the Egyptian culture's emphasis on social support and helping those in need might have influenced students to prioritize social sustainability issues as a cultural duty. Moreover, Egypt has launched the "Decent Life Initiative," targeting 4,500 underprivileged villages to uplift their social conditions and potentially benefit 60 million Egyptians [52]. Therefore, the students in this study may have felt compelled to engage in practical actions to address these challenges despite holding less favorable attitudes.

Interestingly, the students in this study expressed less favorable attitudes while simultaneously demonstrating a higher level of engagement in environmentally sustainable behaviors. This finding aligns with previous studies indicating that nursing students exhibited the highest scores for sustainability behavior in the environmental dimension, followed by the social dimension, while the economic dimension received the lowest scores [36, 50]. The complex and global nature of environmental sustainability issues, particularly those related to climate change and pollution, has received significant attention in both the global and Egyptian media. For instance, the "Go Green Initiative," launched by the Egyptian Ministry of Environment, aims to promote environmental awareness, encourage behavioral change, and urge citizens, especially young people, to protect the environment. This extensive media coverage and initiatives have emphasized the seriousness of environmental issues and the urgent need to address them. Therefore, the students in this study might have felt driven to engage in practical actions to tackle these challenges despite holding less favorable attitudes.

Regarding economic sustainability, there is an apparent knowledge gap and low behavioral engagement among the students across the three universities. This may be attributed to students perceiving economic sustainability as less directly relevant to their future roles as healthcare providers. Thus, they might need to find it more worthy of attention. Similar results have been observed in previous studies conducted in Egypt [36, 37]. This trend is not unique to Egypt, as studies on university students in Spain [53], Sweden [54], and India [49] have reported similar results.

Furthermore, the findings of the present study identified four significant predictors of SC: age, place of residence, family income, and awareness of the SDGs. In line with previous research conducted among college students in different countries [19, 51, 55], older students consistently exhibited higher SC levels than did their younger counterparts. This trend is attributed to older students' increased knowledge and experiences throughout their academic journeys. Therefore, it is no surprise that a significant association was found between students' SC and their academic years. Family income and prior knowledge of the SDGs were identified as predictors of sustainability consciousness. Consistent with earlier research, students from families with sufficient income [56, 57] and those with prior knowledge of the SDGs [40, 58] demonstrated higher sustainability consciousness levels than did their peers.

Regarding the place of residence, students in rural areas showed greater SC than did their urban counterparts. Although this finding differs from that of Akhter & Malaviya (2015) [59], it is broadly consistent with that of El-Hamed et al. (2022), who found that students in rural areas had significantly higher attitude scores toward environmental goals than did those in urban areas [51]. Rural areas may provide students with a direct connection to nature, fostering daily interactions with ecosystems that deepen their environmental appreciation and understanding of sustainability. Conversely, urban areas often contend with higher industrialization and urbanization.

The variation in place of residence likely contributed to the significant variations observed among the students from the three universities. Specifically, nursing students at the university demonstrated the highest median scores across all SC constructs, dimensions, and total SC scores, whereas students at Alexandria University showed the lowest median scores. The geographical, cultural, and socioeconomic context in which the university is situated likely played a role in this pattern. University in Upper Egypt is characterized by a predominantly rural landscape, and certain areas within this region lack access to essential resources such as water and electricity. These circumstances may drive residents, including students, to adopt sustainable consumption practices, fostering a culture of sustainability. Additionally, initiatives focused on poverty alleviation and sustainability promotion in rural Upper Egypt may have played a significant role in shaping the SC of the nursing students at Sohag University regarding sustainable practices.

Sohag University's commitment to sustainability is evident in its global ranking as the 400th most sustainable university worldwide in 2021, while Alexandria University is ranked slightly lower at 432nd place. In contrast, Damanhour University must still be classified in green university rankings. Moreover, in 2022, Sohag university was recognized as one of Egypt's most environmentally sustainable universities [60, 61]. It strongly emphasizes sustainability initiatives and maintaining a green environment. These efforts include implementing various sustainability seminars, activities (e.g., tree planting), and programs to raise environmental awareness and ensure proper waste disposal [62]. Previous studies have indicated that students' participation in sustainable development activities positively influences their SC [51, 63].

## Limitations of the study

This study has several strengths, as it offers insight into an underexplored demographic: Egyptian nursing students. The utilization of validated questionnaires and a substantial, randomly selected sample from three universities enhances the credibility of the findings. However, the study's exclusive focus on these universities limits its generalizability to broader populations or other countries. The unique cultural, geographical, and socioeconomic characteristics of these institutions may constrain the transferability of the results to different contexts. To enhance generalizability, future research could consider a more diverse sample, including faculty, staff, and alumni, and compare sustainability consciousness and practices across various Egyptian universities with those from diverse international settings. Another limitation stems from the reliance on self-reported measures, introducing the potential for response biases such as social desirability or recall bias. Employing objective measures or observational methods could augment the robustness of the findings.

Additionally, the cross-sectional design provides a snapshot of the situation but needs to catch up in tracking changes over time or establishing causality. To mitigate this limitation, longitudinal studies could be implemented. Despite these limitations, this study significantly contributes to the expanding body of research on sustainability issues.

## Conclusion

This study highlighted the sustainability consciousness among nursing students at three Egyptian universities. The study also highlighted the prevalent reliance on social media for information among nursing students across these universities, raising concerns about potential gaps in sustainability coverage within academic settings. This underscores the pivotal role of academic institutions in delivering comprehensive sustainability education, including integrating the SDGs into nursing curricula, particularly as a significant proportion of students in this study needed more awareness about the SDGs. Compared to Alexandria and Damanhour universities, Sohag University's notable commitment to sustainable extracurricular activities and practices has contributed to the greater SC of its students across social, environmental, and economic dimensions. Furthermore, the study identified age, place of residence, family income, and familiarity with the SDGs as consistent predictors of sustainability consciousness.

## Implications of the study

The findings of this study hold significant implications across various domains including research, education, and practice. Firstly, Egyptian universities are urged to integrate Sustainable Development Goals (SDGs) into their nursing curricula, offering culturally sensitive sustainability education to tackle both local and global sustainability challenges effectively. Collaboration between policymakers and educators is imperative to develop interdisciplinary sustainability modules that foster holistic comprehension of sustainability, emphasizing the interconnectedness of environmental, social, and economic dimensions. Nursing programs should incorporate hands-on experiences like clinical placements and projects to facilitate the implementation of sustainable practices among students. Moreover, community engagement initiatives can be instrumental in addressing sustainability issues, allowing students to apply their knowledge in real-world scenarios. It is essential to provide comprehensive training for nursing educators to effectively impart sustainability concepts, thereby fostering a more sustainability-conscious mindset among students. Policymakers can facilitate knowledge dissemination by sharing successful strategies and case studies in sustainability education and healthcare sustainability with universities. In terms of research, identifying educational gaps in nursing curricula, conducting longitudinal studies to track the evolution of students' sustainability consciousness, and undertaking comparative research across different regions or countries are crucial avenues for advancing our understanding of sustainability in nursing education and practice.

### Supplementary Information

Additional file 1.

## Data Availability

All data generated or analyzed during this study are included in this published article and its supplementary information files.
