# Correction: Sustainability consciousness among nursing students in Egypt: a cross-sectional study

**DOI:** 10.1186/s12912-024-02148-9

**Published:** 2024-07-18

**Authors:** Marwa Ahmed El-Sayed Mohamed, Eman Ghallab, Ragaa Abdullah Ahmed Hassan, Shaimaa Mohamed Amin

**Affiliations:** 1 Community Health Nursing Department, Faculty of Nursing, Alexandria University, Alexandria, Egypt; 2 Nursing Education Department, Faculty of Nursing, Alexandria University, Alexandria, Egypt; 3 Family and Community Health Nursing Department, Faculty of Nursing, Sohag University, Sohag, Egypt; 4 Community Health Nursing Department, Faculty of Nursing, Damanhour University, Damanhour, Egypt

**Correction to: BMC Nursing (2024) 23:343**

10.1186/s12912-024-01990-1

Following publication of the original article [1], the authors reported an error in the display order of the author names. The correct author names have been provided in this Correction.

The original article [1] has been corrected.
